# Faecal immunoglobulin A as a non-invasive biomarker of mucosal immunity and health in zoo and wild mammals

**DOI:** 10.1093/conphys/coag054

**Published:** 2026-07-28

**Authors:** Worapong Kosaruk, Patcharapa Towiboon, Sarisa Klinhom

**Affiliations:** Faculty of Veterinary Medicine, Chiang Mai University, 155 Moo 2, Mae Hia, Mueang Chiang Mai, Chiang Mai 50100, Thailand; Elephant, Wildlife, and Companion Animals Research Group, Chiang Mai University, 239 Huay Kaew Road, Suthep, Mueang Chiang Mai, Chiang Mai 50200, Thailand; Faculty of Veterinary Medicine, Chiang Mai University, 155 Moo 2, Mae Hia, Mueang Chiang Mai, Chiang Mai 50100, Thailand; Elephant, Wildlife, and Companion Animals Research Group, Chiang Mai University, 239 Huay Kaew Road, Suthep, Mueang Chiang Mai, Chiang Mai 50200, Thailand; Faculty of Veterinary Medicine, Chiang Mai University, 155 Moo 2, Mae Hia, Mueang Chiang Mai, Chiang Mai 50100, Thailand; Elephant, Wildlife, and Companion Animals Research Group, Chiang Mai University, 239 Huay Kaew Road, Suthep, Mueang Chiang Mai, Chiang Mai 50200, Thailand

**Keywords:** Faecal immunoglobulin A, mucosal immunity, non-invasive biomarker, wildlife health, zoo animals

## Abstract

Non-invasive biomarkers of immune function are increasingly important for assessing health, welfare and disease risk in zoo and wild mammals, particularly because they support repeated monitoring while minimizing handling-related disturbance. Secretory immunoglobulin A (IgA), a key component of mucosal immunity, can be quantified from faecal samples and provides a practical measure of gut-associated immune activity without invasive sampling. We conducted a systematic literature review of 21 peer-reviewed studies that quantified faecal IgA across diverse mammalian taxa and ecological contexts. Across species, faecal IgA was technically measurable and biologically responsive, but its interpretation was strongly context dependent. Reported patterns reflected interactions among pathogen exposure, physiological stress, nutritional state, life-history stage and management conditions. In captive settings, faecal IgA frequently varied with individual heterogeneity and management factors and showed inconsistent alignment with endocrine stress markers. In free-ranging populations, faecal IgA more commonly tracked parasite burden, reproductive investment, seasonal variation and host–microbiome dynamics. However, most ecological and welfare-associated patterns were derived from observational designs, which limit causal inference. Additionally, methodological heterogeneity in assay validation, sample processing and preservation limited direct quantitative comparison among studies. Overall, faecal IgA does not function as a unidimensional indicator of stress or welfare, but rather as a context-sensitive marker of mucosal immune allocation. We integrate these findings into a conceptual framework linking external pressures, mucosal immune dynamics, complementary biomarkers and health-related outcomes to guide interpretation across zoo and wild settings. When embedded within longitudinal and multi-marker approaches supported by species-specific validation, faecal IgA has potential to contribute meaningfully to non-invasive health assessment in conservation physiology.

## Abbreviations

ACTH: adrenocorticotropic hormone
EEHV: elephant endotheliotropic herpesvirus
ELISA: enzyme-linked immunosorbent assay
HPA: hypothalamic–pituitary–adrenal
IgA: immunoglobulin A

## Introduction

Mucosal surfaces represent the primary interface between mammals and their environment and are continuously exposed to dietary antigens, commensal microbiota and pathogenic organisms. Effective mucosal immunity is therefore essential for maintaining physiological homeostasis and preventing disease. Among the immune components operating at these surfaces, secretory immunoglobulin A (IgA) plays a central role as the dominant antibody isotype in the gastrointestinal tract (Mantis *et al.*, 2011; Corthesy, 2013; Celi *et al.*, 2019; Pietrzak *et al.*, 2020). By binding luminal antigens and microorganisms, IgA limits epithelial invasion, modulates microbial composition and contributes to immune tolerance, positioning it as a cornerstone of intestinal immune regulation (Macpherson *et al.*, 2008; Mantis *et al.*, 2011; Planer *et al.*, 2016). The production of high-affinity IgA is crucial for maintaining the symbiotic relationship with the gut microbiome and is a key determinant of barrier integrity, preventing dysbiosis-driven inflammation (Bunker and Bendelac, 2018; Li *et al.*, 2020; Pannoni *et al.*, 2022). Because mucosal immune activity integrates signals from infection, nutrition and physiological stress, markers of IgA production can provide insight into immune resource allocation, referring to how animals distribute energetic and physiological resources among maintenance, growth, reproduction, stress responses and immune defence under varying environmental conditions. As the primary immunoglobulin isotype at the mucosal surface, IgA is produced predominantly by plasma cells located within mucosal-associated lymphoid tissues, including the lamina propria of the gastrointestinal tract. Dimeric IgA is subsequently transported across epithelial cells via the polymeric immunoglobulin receptor and released into the lumen as secretory IgA, where it exerts its immune functions (Johansen and Kaetzel, 2011; Staley *et al.*, 2018).

Importantly, IgA can be measured non-invasively through faecal samples, providing a practical window into gut-associated mucosal immunity without the need for blood collection or invasive procedures. Faecal IgA has been utilized as an indicator of immune function and disease risk across a range of mammalian systems, including humans (Planer *et al.*, 2016) and various domestic and wild mammals (Watt *et al.*, 2016; Staley *et al.*, 2018; Zannoni *et al.*, 2020; Burton *et al.*, 2025). In these contexts, secretory IgA represents the mucosal antibody involved in local immune defence, whereas faecal IgA serves as its non-invasive proxy recovered from faeces. This marker has been linked to gastrointestinal disease, stress-related immune modulation and clinical outcomes, supporting its relevance as a biologically meaningful indicator of gut-associated immunity. Specifically, recent reviews have highlighted how faecal IgA concentrations fluctuate in response to both acute and chronic stressors, making it a valuable tool for welfare monitoring in species where handling or restraint is undesirable or impractical (Staley *et al.*, 2018; Kosaruk *et al.*, 2020; Zannoni *et al.*, 2020).

In zoo and wild settings, the potential value of faecal IgA extends beyond disease diagnostics. Conservation physiology increasingly emphasizes the integration of physiological biomarkers to understand how animals respond to ecological stressors, anthropogenic disturbance and management practices (Madliger *et al.*, 2018). The need for reliable, non-invasive physiological indicators to assess health and welfare in threatened and managed species is a persistent challenge in conservation efforts (Ohmer *et al.*, 2021). Because mucosal immunity lies at the intersection of environmental exposure, energetic balance and physiological stress, faecal IgA has direct relevance to assessing disease susceptibility, welfare state and ecological resilience within the emerging field of ecoimmunology (Macpherson *et al.*, 2008; Staley *et al.*, 2018; Ohmer *et al.*, 2021). Unlike endocrine markers that primarily reflect hypothalamic–pituitary–adrenal (HPA) axis activation, IgA captures immune investment at the mucosal interface, where nutritional status, pathogen exposure and chronic stress converge (Campos-Rodríguez *et al.*, 2013; Staley *et al.*, 2018).

Despite growing interest in faecal IgA as a non-invasive immune marker, its application in conservation and wildlife research remains fragmented. Existing studies are typically species-specific and vary widely in ecological setting, assay methodology and interpretive framework. This variation in methodology, particularly regarding sample preparation, assay validation and standardization of results across taxa, poses a significant obstacle to cross-species comparison (Staley *et al.*, 2018). While faecal IgA has been measured in a limited number of zoo-housed and wild mammals, there is currently no cross-species synthesis that integrates these findings across taxa or ecological contexts. Moreover, the absence of a unified interpretive framework has limited the translation of faecal IgA from a promising physiological measure into a broadly applicable tool for conservation physiology. Previous reviews have largely addressed IgA biology in humans, model organisms, domestic animals or broader welfare contexts (Corthesy, 2013; Staley *et al.*, 2018; Ohmer *et al.*, 2021), leaving a gap in cross-species synthesis for zoo and wild mammals where non-invasive monitoring is especially relevant.

Here, we address this gap by systematically reviewing peer-reviewed studies that have quantified faecal IgA in zoo and wild mammals and by synthesizing their findings within an integrative conceptual framework. Our objectives are to evaluate how faecal IgA has been applied across species and contexts, to identify methodological and biological factors influencing its interpretation and to propose a suggestive framework for using faecal IgA as a context-dependent indicator of mucosal immunity, health and welfare in conservation physiology. By consolidating existing evidence and clarifying interpretive boundaries, this review aims to provide a foundation for future research and applied monitoring that incorporates faecal IgA into multi-biomarker approaches for assessing mammalian health in both managed and natural environments.

## Materials and Methods

### Search strategy and information sources

We conducted a systematic literature search following PRISMA 2020 guidelines (Page *et al.*, 2021) to identify peer-reviewed studies that measured faecal IgA in mammals in zoological settings, sanctuaries, conservation breeding programmes, semi-wild or free-ranging contexts. Searches were performed in PubMed, Scopus, Embase and Google Scholar, covering the period from January 2014 to September 2025. This timeframe was selected to focus on contemporary applications of non-invasive immunological biomarkers in conservation physiology and animal welfare research, particularly faecal-based immune biomarkers. The final search was completed on 12 September 2025.

Full database-specific search strings and filters are provided in Supplementary Appendix S1. Searches combined IgA-related terms such as ‘immunoglobulin A’, ‘IgA’, ‘secretory IgA’, ‘faecal immunoglobulin’ and ‘faecal IgA’ with broad mammalian and wildlife-related terms, including ‘mammal’, ‘primate’, ‘carnivore’, ‘ungulate’, ‘zoo’, ‘captive’ and ‘wildlife’. Species-specific keywords were added to increase relevance. These taxonomic terms were used to increase search sensitivity and capture relevant mammalian studies, but were not used to restrict eligibility to predefined taxa. The search strategy was intentionally broad and not limited to specific taxa, allowing us to capture studies where faecal IgA appeared opportunistically in diverse species.

All retrieved records were exported into a single database. Duplicates were removed using automated filtering in Zotero and manual review. After automated and 133 manual deduplication, 17 547 unique records remained. Reference lists of relevant articles were checked opportunistically to ensure that no eligible studies were missed.

### Eligibility criteria

Studies were included if they met all of the following criteria.

(1) The study involved mammals in zoo settings, sanctuaries, conservation breeding programmes, semi-wild or free-ranging contexts.
(2) Faecal IgA was quantified directly. Eligible forms included total IgA, secretory IgA and pathogen-specific IgA.
(3) The article presented primary research in any form, including observational studies, longitudinal monitoring, experimental or intervention work, assay validation papers, short communications and field-based disease surveillance reports.
(4) Outcomes were relevant to mucosal immunity, gastrointestinal health, parasitism, infection, disease detection, welfare assessment or stress physiology.

Studies were excluded if they involved humans, laboratory rodents, domestic livestock (e.g. cattle, pigs, poultry), companion animals (e.g. dogs, cats, horses) or fish. Articles were also excluded if IgA was measured only in serum or saliva, or if the work focused on *in vitro* systems, molecular engineering or purely biomedical clinical conditions. Non-research formats such as reviews or commentaries were removed.

### Screening process

**Figure 1:**
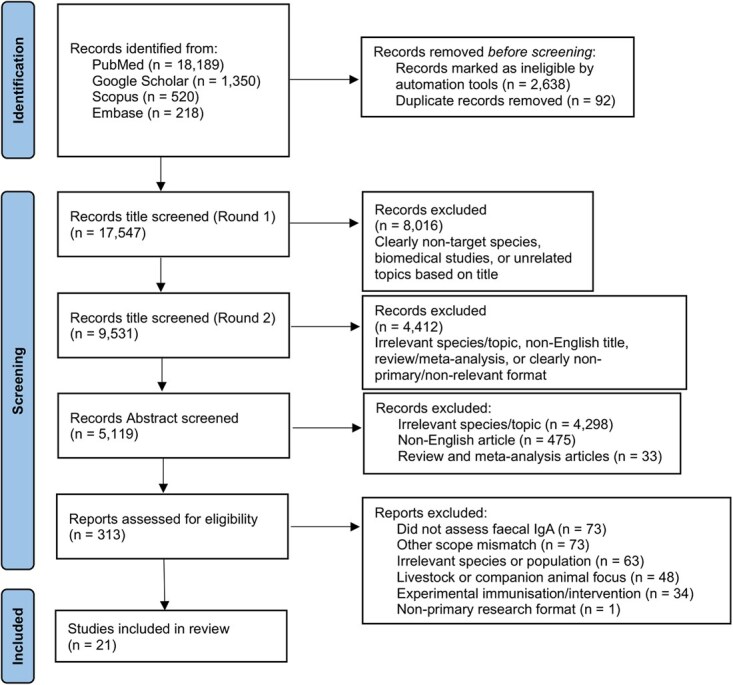
PRISMA flow diagram illustrating the literature search, screening, eligibility assessment and study inclusion process for the literature review of faecal IgA in zoo and wild mammals (January 2014 to September 2025).

Screening and study selection followed PRISMA 2020 guidelines, with the full screening pathway summarized in the PRISMA flow diagram (Fig. 1). The screening process was conducted in multiple stages and overseen by the lead reviewer, with ambiguous cases discussed among co-authors to ensure consistent application of eligibility criteria.

Records identified through database searching were first subjected to an initial title screening to remove clearly irrelevant articles, including non-mammalian studies, biomedical or laboratory-focused research and articles unrelated to faecal IgA based on title alone. This step was supported by automated text-based filtering tools to facilitate efficient removal of obviously ineligible records.

All remaining records then underwent manual title and abstract screening by the reviewer to further exclude studies that did not meet the predefined inclusion criteria. Full-text assessment was subsequently conducted for all potentially eligible articles to confirm study design, target population and relevance to mucosal immunity in zoo or wild mammals.

The most common reasons for exclusion at the full-text stage were the absence of faecal IgA measurements, mismatch with the target scope and a primary focus on livestock or companion animals outside a conservation framework. Following full-text evaluation, 21 studies met all inclusion criteria and were retained for final synthesis.

### Data extraction and synthesis

For each included study, we extracted information into a standardized data matrix. Extracted variables included species, ecological or husbandry setting, type of IgA assay, sample processing and preservation technique, study design, co-measured physiological markers and the primary research question. We also recorded key outcomes concerning faecal IgA, such as direction of change, sources of variation and reported associations with stress physiology, parasite burden, gastrointestinal health, seasonality, reproductive status or disease events.

Because the included studies varied widely in taxon, assay type, outcome definition and research design, a meta-analysis was not feasible. Instead, after extraction, findings were synthesized qualitatively. The synthesis involved grouping studies according to shared methodological features and biological themes, which allowed consistent comparison across diverse species and contexts. We therefore applied a physiology-informed validation appraisal framework focusing on four domains: (i) analytical validation (e.g. parallelism, spike-and-recovery, assay precision), (ii) biological validation [e.g. adrenocorticotropic hormone (ACTH) challenge or defined physiological perturbation], (iii) documentation of storage and sample stability testing, and (iv) study design strength (experimental or longitudinal versus cross-sectional).

Based on these indicators, studies were classified into three qualitative evidence categories: high, moderate or preliminary validation strength. This appraisal was used to contextualize interpretation of reported IgA associations rather than to exclude studies from synthesis.

## Results

### Characteristics of included studies

The 21 studies included in this review spanned a wide taxonomic and ecological range, illustrating the emerging applicability of faecal IgA as a non-invasive marker of mucosal immunity across mammalian systems. The taxonomic scope, ecological context and study designs of the included literature are summarized in Table 1.

**Table 1:** Overview of included studies measuring faecal IgA in zoo and wild mammals

**ID**	**Author (year)**	**Species**	**Context**	**Sample size**	**Study aim**	**Other biomarkers measured**
1	Huang *et al.* (2014)	Sichuan golden monkey (*Rhinopithecus roxellana*)	Captive; Shanghai Wildlife Zoo, China	6 adults (3 males and 3 females); repeated faecal sampling across 4 seasons	Examine seasonal variation in faecal cortisol and immunoglobulins (IgA, IgG and IgM) in relation to physiological status and welfare	Faecal cortisol, IgG and IgM
2	Yin *et al.* (2015)	Reindeer (*Rangifer tarandus*)	Semi-domesticated/free-ranging; Inner Mongolia, China	80 individuals across 4 age classes; 20 individuals per group (10 males and 10 females)	Assess stress and immune physiology in reindeer using faecal cortisol and immunoglobulins across age and sex classes	Faecal cortisol, IgG and IgM
3	Seo *et al.* (2016)	Wild boar (*Sus scrofa coreanus*); domestic pig (*S. scrofa domestica*) and multiple other mammalian species included for assay cross-reactivity comparison	Mixed; managed and multi-species comparison, South Korea	Wild boar: 40 faecal samples; domestic pigs: 50 faecal samples; cross-reactivity panel: 22 species and 12 samples per species	Evaluate cross-reactivity and applicability of commercial porcine and bovine IgA ELISAs for wild boar monitoring	None
4	Yoshida *et al.* (2016)	Bonobo (*Pan paniscus*); captive chimpanzee (*Pan troglodytes*) included for assay validation	Free-ranging; multiple bonobo sites, Democratic Republic of the Congo; captive chimpanzees used for assay validation	Wild bonobos: 98 faecal samples after duplicate removal; captive chimpanzees: samples from 9 individuals for assay validation	Develop a faecal IgA-based serological approach to assess LCV exposure	Serum LCV-specific IgG
5	Lantz *et al.* (2018)	Eastern chimpanzee (*P. troglodytes schweinfurthii*)	Free-ranging; Gombe National Park, Tanzania; 1 captive chimpanzee used for ACTH validation	Wild chimpanzees: 1463 faecal samples from 59 individuals; captive chimpanzee: repeated samples from 1 male for ACTH validation	Develop a faecal IgA assay for wild chimpanzees and assess variation with age, sex, reproductive status, time of day and season	fGCM during ACTH validation
6	Edwards *et al.* (2019)	Asian elephant (*Elephas maximus*)	Captive; Smithsonian’s National Zoo, USA	4 adult females sampled over 6 months; additional faecal samples collected during severe illness in 1 female	Develop an immunoassay to quantify IgA in Asian elephant faeces, saliva, urine and serum	Glucocorticoids across faeces, saliva, urine and serum; IgA across multiple matrices
7	Albery *et al.* (2020)	Red deer (*Cervus elaphus*)	Free-ranging; Isle of Rum, Scotland	837 faecal samples from 140 adult females	Examine relationships among gestation, lactation, parasite burden and faecal total and parasite-specific IgA	Strongyle FEC, *Fasciola*, *Elaphostrongylus*
8	Gesquiere *et al.* (2020)	Baboon (*Papio cynocephalus* with some *Papio anubis* admixture)	Free-ranging; Amboseli, Kenya; small captive subset used for validation and storage tests	Wild baboons: 310 faecal samples from 168 individuals; captive baboons: 4 individuals for assay validation	Validate faecal IgA measurement and examine associations with parasitism and life-history variables	*Trichuris* egg counts, parasite richness
9	Kosaruk *et al.* (2020)	Asian elephant (*E. maximus*)	Captive; tourist camps, Thailand	44 adult females; repeated faecal and salivary sampling	Assess effects of tourism-related activities on glucocorticoids and IgA	fGCM, salivary cortisol
10	Tombak *et al.* (2020)	Domestic donkey (*Equus africanus asinus*), plains zebra (*Equus quagga*) and Grevy’s zebra (*Equus grevyi*)	Mixed; free-ranging zebras and semi-managed donkeys, Mpala landscape, Kenya	Donkeys: 199 samples; Grevy’s zebras: 101 samples; plains zebras: 37 samples	Develop faecal immunoglobulin assays for African equids and examine relationships with parasite infections, body condition and environmental factors	Strongyle FEC, body condition score
11	Behringer *et al.* (2021)	Barbary macaque (*Macaca sylvanus*)	Semi-free-ranging/provisioned; Affenberg Salem, Germany	ACTH challenge: 9 adults with repeated faecal sampling; observational sample size not reported	Evaluate responsiveness of faecal IgA to ACTH-induced HPA axis activation and natural stressors	fGCM
12	Boonprasert *et al.* (2021)	Asian elephant calf (*E. maximus*)	Captive; elephant calves, Thailand	9 calves (3 without prior EEHV, 6 with prior EEHV); repeated sampling over 12 months; 1 fatal EEHV-HD case	Evaluate haematological and biomarker profiles in relation to EEHV infection history and a fatal EEHV-HD case	Salivary cortisol, fGCM, salivary IgA, CBC, EEHV qPCR viral load
13	Ferreira *et al.* (2021)	Spotted hyena (*Crocuta crocuta*)	Free-ranging; Serengeti National Park, Tanzania	174 individuals; repeated faecal sampling; exact sample number not reported	Assess whether faecal immune markers reflect parasite infection and longevity in a wild carnivore	Parasite infection metrics
14	Hall *et al.* (2021)	Chimpanzee (*P. troglodytes*)	Captive; 16 AZA zoos, USA	41 adults; 3226 faecal samples collected over 3 months	Examine relationships among behavioural diversity, fGCM, faecal IgA and abnormal behaviours	fGCM, behavioural indices
15	Li *et al.* (2021)	Forest musk deer (*Moschus berezovskii*)	Captive; musk deer farm, China	15 newborns; 81 faecal samples collected across developmental stages	Characterize intestinal microbiota and faecal cortisol, T3 and IgA from birth to one month after weaning	Faecal cortisol, faecal T3; gut microbiome
16	Guo *et al.* (2023)	Taihangshan macaque (*Macaca mulatta tcheliensis*)	Free-ranging with provisioning; China	89 faecal samples; mating and non-mating periods compared; analytical sample size reported by sex and period	Investigate variation in faecal cortisol and IgA across reproductive periods	Faecal cortisol
17	Pethig *et al.* (2024)	Red-fronted lemur (*Eulemur rufifrons*)	Mixed; zoo population in Germany and free-ranging in Kirindy Forest, Madagascar	Zoo: 2 females, 16 faecal samples; wild: 36 individuals, 422 samples (1–18 samples per individual)	Validate faecal IgA measurement and assess effects of sample handling and storage	None
18	Li *et al.* (2024)	Przewalski’s horse (*Equus ferus przewalskii*)	Reintroduced/free-ranging; Karamaili Mountain Nature Reserve, China	14 individuals (7 healthy, 7 diarrhoeic); 1 faecal sample per individual	Examine microbial diversity, hormones and faecal IgA as indicators of gastrointestinal health in reintroduced animals	Faecal cortisol, faecal T3, gut microbiome
19	Sereme *et al.* (2024)	Western chimpanzee (*P. troglodytes verus*)	Free-ranging; Dindefelo Community Nature Reserve, Senegal; captive zoo chimpanzees used as controls	Wild chimpanzees: 48 faecal samples from 29 individuals; captive chimpanzees: 34 samples from 19 individuals	Investigate treponematosis (caused by *T. pallidum*) using a faecal serology approach targeting total and pathogen-specific immunoglobulins	Pathogen-specific IgG/IgM, *T. pallidum* qPCR
20	Serres-Corral *et al.* (2025)	Lion (*Panthera leo*)	Captive; 4 zoos, Spain	11 adults; 110 faecal samples (5–15 samples per individual)	Develop and validate a faecal IgA immunoassay for lions and explore its utility as a stress biomarker	fGCM
21	Veloso Soares *et al.* (2025)	Spotted hyena (*C. crocuta*)	Free-ranging; Serengeti National Park, Tanzania	199 faecal samples from 158 individuals	Examine associations among faecal IgA, mucin and gut microbiome composition, and host ecological factors	Faecal mucin, gut microbiome

Abbreviations: AZA, Association of Zoos and Aquariums; CBC, complete blood count; EEHV, elephant endotheliotropic herpesvirus; HD, haemorrhagic disease; FEC, faecal egg count; fGCM, faecal glucocorticoid metabolites; qPCR, quantitative polymerase chain reaction; T3, triiodothyronine.

Primates were the most frequently represented group. These included captive and wild chimpanzees and bonobos studied for baseline immune variation, welfare assessment and infectious disease surveillance (Lantz *et al.*, 2018; Yoshida *et al.*, 2016; Hall *et al.*, 2021; Sereme *et al.*, 2024), free-ranging baboons from long-term field projects (Gesquiere *et al.*, 2020), macaques living under semi-free-ranging or natural conditions (Behringer *et al.*, 2021; Guo *et al.*, 2023), captive golden monkeys examined for seasonal biomarker variations (Huang *et al.*, 2014) and red-fronted lemurs included in assay validation and field application studies (Pethig *et al.*, 2024). Together, primate studies encompassed a continuum from controlled zoological settings to complex natural social systems, providing a substantial foundation for evaluating faecal IgA across contrasting ecological contexts.

Ungulates were also well represented and included free-ranging red deer monitored for reproductive trade-offs between immunity and parasitism (Albery *et al.*, 2020), forest musk deer studied within conservation breeding programmes during early development (Li *et al.*, 2021), reindeer assessed across age and sex classes in semi-domesticated northern systems (Yin *et al.*, 2015), Przewalski’s horses evaluated during reintroduction and gastrointestinal disturbance (Li *et al.*, 2024) and African equids examined in relation to parasite burden and body condition (Tombak *et al.*, 2020). These studies collectively highlight the utility of faecal IgA for addressing questions spanning life history, health status and host–parasite dynamics in large herbivores.

Carnivores were less frequently studied but contributed distinct ecological perspectives. Spotted hyenas featured prominently in work linking faecal IgA to parasite infection and immune–microbiome associations in free-ranging populations (Ferreira *et al.*, 2021; Veloso Soares *et al.*, 2025), while captive lions were examined in the context of assay validation and stress-related immune variation under managed conditions (Serres-Corral *et al.*, 2025). Proboscideans were represented by multiple studies on Asian elephants, including assessments of tourism-related management effects (Kosaruk *et al.*, 2020), cross-matrix assay development for faecal IgA (Edwards *et al.*, 2019) and longitudinal monitoring of calves with differing histories of elephant endotheliotropic herpesvirus (EEHV) infection (Boonprasert *et al.*, 2021).

Study settings varied widely, reflecting the adaptability of faecal IgA methodologies to different logistical and ecological constraints. Zoo-based studies primarily addressed welfare, management and disease surveillance questions in elephants, lions and captive chimpanzees (Edwards *et al.*, 2019; Hall *et al.*, 2021; Serres-Corral *et al.*, 2025). Free-ranging systems characterized investigations of macaques, baboons, hyenas, red deer, reindeer and wild boar (Yin *et al.*, 2015; Seo *et al.*, 2016; Yoshida *et al.*, 2016; Albery *et al.*, 2020; Gesquiere *et al.*, 2020; Ferreira *et al.*, 2021). Conservation breeding or semi-wild contexts, such as musk deer facilities and reintroduced equid populations, provided intermediate settings in which both ecological exposure and husbandry conditions influenced IgA patterns (Li *et al.*, 2021, 2024). This diversity of environments enabled comparison across gradients of natural exposure, anthropogenic disturbance and controlled management.

Study designs ranged from cross-sectional assessments to intensive longitudinal and experimental frameworks. Cross-sectional approaches were common in zoo welfare studies, equid parasite surveys and microbiome-associated investigations (Tombak *et al.*, 2020; Hall *et al.*, 2021; Veloso Soares *et al.*, 2025). Longitudinal sampling enabled analysis of seasonal, reproductive and developmental variation in faecal IgA, including macaques and baboons followed across environmental cycles (Gesquiere *et al.*, 2020; Guo *et al.*, 2023) and elephant calves monitored over a full year (Boonprasert *et al.*, 2021). Experimental manipulation was rare but provided key mechanistic insight, particularly through pharmacological activation of the HPA axis in primates (Behringer *et al.*, 2021). Together, the included studies demonstrate the methodological flexibility of faecal IgA and its capacity to inform research on mucosal immunity, disease ecology, host–microbiome interactions and animal welfare across diverse mammalian systems.

### Methodological considerations in faecal IgA quantification

Detailed information on faecal IgA sample processing, extraction procedures and assay validation for each included study is provided in Table 2. Across the included studies, methodological choices had a clear influence on the detectability, stability and interpretability of faecal IgA.

**Table 2:** Summary of faecal IgA processing, extraction and assay validation across included studies

**ID**	**Author (year)**	**Sample processing**	**Extraction method**	**Assay and validation**
1	Huang *et al.* (2014)	Fresh faeces collected after defecation, stored at −20°C, thawed, homogenized and freeze-dried	Freeze-dried faeces extracted in PBS-Tween-20, agitated, centrifuged and supernatants stored frozen	Commercial ELISA; analytical validation included sensitivity, CVs and recovery; no species-specific biological validation
2	Yin *et al.* (2015)	Fresh faecal pellets collected immediately after defecation and stored at −20°C until analysis	Wet faeces homogenized and extracted in PBS-Tween-20, followed by sequential centrifugation and frozen storage of supernatants	Commercial double-antibody sandwich ELISA; triplicate measurements; no species-specific biological validation reported
3	Seo *et al.* (2016)	Fresh faecal samples were collected from habitats, transported at 4°C and processed shortly after collection	Faeces diluted in PBS-Tween-20 with bovine serum albumin, vortexed, centrifuged and clarified supernatants used for analysis	Commercial porcine IgA ELISA kit; triplicate measurements and documented cross-reactivity across multiple mammalian species
4	Yoshida *et al.* (2016)	Fresh faecal samples collected immediately after defecation, preserved in silica gel or frozen, and stored at −80°C	Faeces extracted in protein extraction buffer with protease inhibitors, vortexed, centrifuged and immunoglobulins purified using protein A/G columns	LCV-specific faecal IgA ELISA adapted from EBV VCA and EA IgA assays; validation included serum comparison in captive chimpanzees and western blotting confirmation
5	Lantz *et al.* (2018)	Fresh faecal samples collected after defecation, frozen within 12 h, and extracts stored frozen before shipment	Wet faeces homogenized in PBS, agitated, centrifuged, aliquoted and frozen	Commercial human IgA ELISA; validation included parallelism, recovery, sensitivity, intra- and inter-assay precision, ACTH challenge and storage stability tests
6	Edwards *et al.* (2019)	Faecal samples frozen at −20°C, lyophilized and prepared for extraction	Lyophilized faecal powder extracted in PBS-Tween-20, agitated overnight, sequentially centrifuged, evaporated, reconstituted and stored frozen	Enzyme immunoassay using anti-human IgA antibodies; validation included parallelism, matrix interference testing and intra- and inter-assay precision
7	Albery *et al.* (2020)	Fresh faecal pellets collected immediately after defecation, homogenized, subsampled and frozen at −20°C	Homogenized faeces extracted in PBS with protease inhibitors, incubated briefly, centrifuged and supernatants stored frozen	Total and parasite-specific faecal IgA ELISAs adapted from sheep immunoassays; validation included serial-dilution parallelism, duplicate measurements and assay controls
8	Gesquiere *et al.* (2020)	Fresh faecal samples collected immediately after defecation, homogenized, chilled, frozen, freeze-dried and stored at −20°C	Freeze-dried faecal powder extracted in IgA diluent, vortexed, centrifuged, clarified and stored at −20°C	Commercial monkey IgA ELISA and a custom in-house ELISA; validation included parallelism, accuracy, matrix testing, intra- and inter-assay precision and assay comparison
9	Kosaruk *et al.* (2020)	Faecal samples stored at −20°C before processing; salivary samples collected using cotton swabs	Faecal samples dried, pulverized and extracted in buffered solution following established elephant protocols by Edwards *et al.* (2019) with some modification	Faecal and salivary IgA enzyme immunoassays previously validated for elephants by Edwards *et al.* (2019); validation procedures revalidated
10	Tombak *et al.* (2020)	Fresh faecal samples collected shortly after defecation, homogenized, subsampled on the day of collection, and stored frozen	Faeces extracted in PBS with protease inhibitor, incubated on ice, centrifuged and supernatants stored frozen	Modified sandwich ELISA adapted from Soay sheep protocols; dilution optimization, duplicate measurements, plate and column effects and repeatability criteria applied
11	Behringer *et al.* (2021)	Faecal samples collected before and after ACTH challenge and during natural stressor observations, then stored frozen	Faecal samples homogenized and extracted using previously established primate faecal IgA protocols by Gesquiere *et al.* (2020) with adaptation	ELISA based on established primate faecal IgA protocols by Gesquiere *et al.* (2020); physiological validation included ACTH challenge and parallel assessment with fGCM
12	Boonprasert *et al.* (2021)	Faecal samples collected at regular intervals, transported chilled and stored at −20°C	Faecal material extracted using established elephant faecal IgA protocols by Edwards *et al.* (2019) and Kosaruk *et al.* (2020)	Enzyme immunoassay adapted from validated elephant IgA methods by Edwards *et al.* (2019) and Kosaruk *et al.* (2020)
13	Ferreira *et al.* (2021)	Fresh faecal samples collected from individually known hyenas and stored frozen before processing	Faecal material homogenized in extraction buffer, centrifuged, clarified and stored frozen	Total faecal IgA enzyme immunoassay previously validated for carnivore faeces; assay performance revalidated
14	Hall *et al.* (2021)	Daily faecal samples collected, frozen at −20°C at each institution, shipped on dry ice and stored frozen	Faecal material extracted using established chimpanzee faecal IgA protocols by Lantz *et al.* (2018)	Commercial human IgA ELISA; analytical validation included parallelism and recovery; physiological validation referenced from prior ACTH challenge studies in chimpanzees by Lantz *et al.* (2018)
15	Li *et al.* (2021)	Fresh faecal samples stored at −80°C, dried to determine water content, and processed for IgA analysis	Faeces extracted in PBS, sequentially centrifuged and supernatants stored at −20°C	Commercial bovine IgA ELISA kit; triplicate measurement and analytical precision reported; no species-specific biological validation described
16	Guo *et al.* (2023)	Fresh faecal samples immediately frozen at −20°C in the field, transported on dry ice within 5 h, vacuum freeze-dried and ground	Freeze-dried faecal powder extracted in PBS-Tween-20, vortexed, incubated, centrifuged twice and stored at −20°C	Commercial monkey IgA sandwich ELISA; duplicate measurements and kit CVs < 20%; species-specific analytical validation (e.g. parallelism) not reported
17	Pethig *et al.* (2024)	Fresh faecal samples homogenized and stored at −20°C; room-temperature storage, long-term freezing and effects of freeze–thaw cycles tested	Faecal material extracted in PBS-Tween using either standard laboratory or simplified field-friendly protocols; supernatants stored frozen	In-house sandwich enzyme immunoassay using rabbit anti-human IgA antibodies; validation included parallelism, accuracy, matrix interference, assay stability and storage effects
18	Li *et al.* (2024)	Fresh faeces collected immediately after defecation, transported in liquid nitrogen and stored at −80°C	Faeces homogenized in PBS, sequentially centrifuged and supernatants stored frozen	Commercial ELISA kit; validation included parallelism, accuracy testing and intra- and inter-assay CVs
19	Sereme *et al.* (2024)	Fresh faecal samples placed directly into extraction buffer with protease inhibitors, transported on dry ice and stored at −80°C	Faecal samples incubated in PBS-based extraction buffer, vortexed, centrifuged, lyophilized, reconstituted and concentrated	Total faecal IgA quantified using commercial ELISA kits; *T. pallidum*-specific IgA measured using adapted ELISA and confirmed by automated Western blotting
20	Serres-Corral *et al.* (2025)	Fresh faecal samples dried at 60°C, ground to powder, sieved and stored at −20°C	Faecal powder extracted in PBS-Tween-20, vortexed, sequentially centrifuged and supernatants used for analysis	Commercial IgA enzyme immunoassay validated for lion faeces; validation included precision, dilution linearity, spike-and-recovery accuracy and assay specificity
21	Veloso Soares *et al.* (2025)	Lyophilized faecal samples homogenized and stored frozen before extraction	Freeze-dried faecal material extracted in saline buffer with protease inhibitors and centrifuged to obtain supernatants	Sandwich ELISA previously validated for spotted hyena faeces by Ferreira *et al.* (2021); duplicate measurements and quality-control criterion of CV < 5%

Abbreviations: CV, coefficient of variation; EA, early antigen; EBV, Epstein–Barr virus; fGCM, faecal glucocorticoid metabolites; IgG, immunoglobulin G; PBS, phosphate-buffered saline; VCA, viral capsid antigen.

Variation was evident from the level of assay selection. Researchers employed a wide range of commercial and laboratory-developed enzyme-linked immunosorbent assay (ELISA) platforms that differed in antibody specificity, target IgA fraction and validation depth. Commercial kits were frequently used across primates, ungulates and suids, and work in wild boar demonstrated that porcine or bovine IgA ELISAs could detect faecal IgA from free-ranging animals with acceptable cross-reactivity (Seo *et al.*, 2016). In contrast, several studies developed or adapted in-house assays for wildlife matrices, including baboons, elephants, lemurs, hyenas and lions, often to improve analytical performance or species relevance (Edwards *et al.*, 2019; Gesquiere *et al.*, 2020; Pethig *et al.*, 2024; Serres-Corral *et al.*, 2025; Veloso Soares *et al.*, 2025). Direct comparison of commercial and laboratory-developed assays in baboons revealed strong correlations but systematic differences in absolute concentrations, indicating that assay choice defines the quantitative scale of faecal IgA rather than altering underlying biological patterns (Gesquiere *et al.*, 2020).

Studies also differed in the form of IgA measured. Most quantified total IgA, whereas others targeted secretory IgA or pathogen-specific IgA, such as lymphocryptovirus (LCV)-specific IgA in bonobos and chimpanzees or *Treponema pallidum*-specific IgA in wild chimpanzees (Yoshida *et al.*, 2016; Sereme *et al.*, 2024). These distinctions reflected different biological questions, ranging from general mucosal immune activity to disease surveillance, but they complicate cross-study comparison because total, secretory and antigen-specific IgA do not necessarily respond in parallel to environmental or physiological stimuli.

Differences in extraction procedures introduced an additional source of heterogeneity. Most studies homogenized faecal material in phosphate-buffered saline or comparable solutions, sometimes with detergents or protease inhibitors, although exact formulations were not consistently reported. Work in elephants, where IgA was quantified across faeces, saliva, urine and serum, emphasized the need to optimize extraction conditions to achieve acceptable parallelism and recovery across individuals and matrices (Edwards *et al.*, 2019). In musk deer and Przewalski’s horses, standardized homogenization protocols supported simultaneous measurement of IgA alongside endocrine and metabolic biomarkers, demonstrating that commonly used extraction approaches can be integrated into multi-biomarker frameworks (Li *et al.*, 2021, 2024). Nevertheless, similarities in extraction strategy do not imply equivalence across studies, and absolute IgA values remain method dependent.

Sample storage and preservation emerged as particularly influential determinants of data quality. Experimental evaluation in wild baboons showed that ethanol preservation markedly reduced IgA detectability, whereas short-term cooling or freezing maintained antibody integrity (Gesquiere *et al.*, 2020). Complementary evidence from red-fronted lemurs demonstrated that storage duration and whether extraction occurred on fresh or frozen material influenced measured secretory IgA concentrations (Pethig *et al.*, 2024). Storage conditions influenced measured IgA concentrations in several studies (Edwards *et al.*, 2019; Hall *et al.*, 2021; Li *et al.*, 2021; Serres-Corral *et al.*, 2025).

Assay validation procedures contributed substantially to confidence in faecal IgA measurements. Parallelism and spike-and-recovery tests were reported in several key studies, including work in baboons, lemurs, elephants, hyenas and chimpanzees, confirming that faecal matrices did not substantially inhibit antibody binding and that assays behaved predictably across serial dilutions (Lantz *et al.*, 2018; Edwards *et al.*, 2019; Gesquiere *et al.*, 2020; Pethig *et al.*, 2024; Veloso Soares *et al.*, 2025). Other studies relied on previously validated protocols or physiological challenges, such as ACTH administration in macaques, to support assay performance (Behringer *et al.*, 2021). Where reported, intra- and inter-assay coefficients of variation (CVs) were within acceptable limits, supporting the technical feasibility of faecal IgA quantification across diverse mammalian species.

Normalization approaches also differed among studies. Some quantified IgA relative to wet faecal mass, whereas others used dried or lyophilized material, as in elephant assay development and several primate studies (Yoshida *et al.*, 2016; Edwards *et al.*, 2019; Pethig *et al.*, 2024). Although both approaches yielded biologically interpretable results within studies, they are not directly interchangeable without accounting for moisture content and matrix composition. In addition, substantial inter-individual variation in faecal IgA was reported even among animals sharing similar environments or management conditions (Kosaruk *et al.*, 2020; Hall *et al.*, 2021), suggesting that biological heterogeneity is amplified by matrix effects and sampling variability.

A smaller subset of studies highlighted temporal sources of variation. In wild baboons, diurnal patterns and irregular feeding schedules contributed to short-term fluctuation in faecal IgA (Gesquiere *et al.*, 2020), whereas species with long gut transit times, such as elephants, appeared to exhibit more temporally smoothed profiles (Edwards *et al.*, 2019; Kosaruk *et al.*, 2020; Boonprasert *et al.*, 2021). These findings indicate that temporal variation was reported in a subset of studies and should be considered when comparing repeated faecal IgA measurements.

### Methodological validation strength

The methodological validation profile of included studies is summarized in Table 3. Of the 21 studies, four incorporated both analytical and biological validation procedures, including physiological perturbation or comprehensive assay characterization, and were therefore classified as high validation strength. These studies provide the most direct evidence that faecal IgA responds to defined physiological activation rather than reflecting assay artefact or uncontrolled environmental variation. Ten studies reported species-specific analytical validation, such as parallelism, recovery or precision testing, but did not include experimental biological challenge. These were categorized as moderate validation strength. The remaining seven studies primarily applied previously validated immunoassays without reporting new analytical validation within the study itself and were therefore classified as preliminary. Notably, experimental evidence for acute IgA responsiveness was derived almost exclusively from high-validation studies. In contrast, many reported associations linking faecal IgA to ecological context, life-history stage or chronic management conditions originated from moderate or preliminary designs. Accordingly, directional patterns across taxa were interpreted in relation to validation depth and study design, particularly for claims involving chronic stress, immune suppression or welfare-related effects.

**Table 3:** Methodological validation strength of faecal IgA measurement across included studies

**ID**	**Author (year)**	**Analytical validation summary**	**Biological validation**	**Storage/stability assessment**	**Study design**	**Evidence strength** ^**a**^
1	Huang *et al.* (2014)	Sensitivity, CVs, recovery (commercial ELISA)	No	Not reported	Seasonal observational	Moderate
2	Yin *et al.* (2015)	Commercial double-antibody sandwich ELISA; triplicate only	No	Not reported	Cross-sectional observational	Preliminary
3	Seo *et al.* (2016)	Commercial porcine IgA ELISA; triplicate; cross-reactivity documented	No	Not reported	Cross-sectional methodological	Preliminary
4	Yoshida *et al.* (2016)	ELISA adaptation and Western blot confirmation	Indirect; serum concordance	Not reported	Cross-sectional assay adaptation	Moderate
5	Lantz *et al.* (2018)	Parallelism, recovery, sensitivity, intra- and inter-assay precision	Yes; ACTH challenge	Yes; stability tests	Longitudinal with experimental component	High
6	Edwards *et al.* (2019)	Parallelism, matrix testing, intra- and inter-assay precision	No	Not explicitly tested	Assay development; multi-matrix methodological	Moderate
7	Albery *et al.* (2020)	Parallelism, duplicate controls	No	Not reported	Longitudinal field observational	Moderate
8	Gesquiere *et al.* (2020)	Parallelism, accuracy, assay comparison, intra- and inter-assay precision	No	Partial; preservation effects tested	Longitudinal field with methodological component	Moderate
9	Kosaruk *et al.* (2020)	Parallelism, recovery, sensitivity, intra- and inter-assay precision	No	Not reported	Repeated-measures observational	Moderate
10	Tombak *et al.* (2020)	Dilution optimization, duplicate measurement, repeatability assessment	No	Not reported	Field observational with methodological component	Moderate
11	Behringer *et al.* (2021)	Parallelism, intra- and inter-assay precision, extraction optimization	Yes; ACTH challenge	Storage comparison reported	Experimental and observational	High
12	Boonprasert *et al.* (2021)	Referenced prior validation; duplicate measurement	No	Not reported	Longitudinal monitoring	Preliminary
13	Ferreira *et al.* (2021)	Assay modified and validated; analytical performance reported	Ecological association only	Not reported	Observational field	Moderate
14	Hall *et al.* (2021)	Referenced prior validation	No	Not reported	Multi-institution observational	Preliminary
15	Li *et al.* (2021)	Intra- and inter-assay precision reported	No	Not reported	Longitudinal observational	Preliminary
16	Guo *et al.* (2023)	Kit CV reported; duplicate measurement	No	Not reported	Observational field	Preliminary
17	Pethig *et al.* (2024)	Parallelism, matrix, interference, intra- and inter-assay precision	No	Yes; storage duration and freeze–thaw tests	Methodological with field component	High
18	Li *et al.* (2024)	Parallelism, accuracy	No	Not reported	Cross-sectional observational	Moderate
19	Sereme *et al.* (2024)	Specific IgA ELISA and Western blot confirmation	Indirect; serum concordance	Not reported	Cross-sectional, pathogen-specific assay application	Moderate
20	Serres-Corral *et al.* (2025)	Parallelism, linearity, spike-and-recovery, intra- and inter-assay precision; species-specific validation	No	Not reported	Assay validation with short-term longitudinal application	High
21	Veloso Soares *et al.* (2025)	Referenced prior species validation; duplicate measurement; CV threshold applied	No	Not reported	Multi-year longitudinal field observational	Preliminary

Preliminary = assay application based primarily on commercial kits or previously validated protocols, with limited or no new analytical validation reported within the study.

a ^a^Evidence strength classification: high = species-specific analytical validation plus biological validation or comprehensive assay characterization, such as physiological challenge or formal stability testing. Moderate = species-specific analytical validation, such as parallelism, recovery, accuracy or precision testing, reported within the study, without biological challenge.

### Captive mammals: welfare, management and stress–immune dynamics

Reported associations between faecal IgA and biological or ecological factors across captive and free-ranging mammals are synthesized in Table 4. In captive studies, faecal IgA variation reflected a combination of management context, environmental or social demands and individual physiological state, rather than aligning consistently with a single dimension of stress or welfare.

**Table 4:** Factors associated with variation in faecal IgA across included studies

**Factor**	**Direction of association with faecal IgA**	**Context, species and evidence from included studies**
Age/life stage	Age-structured; non-linear	Semi-domesticated/free-ranging reindeer: faecal IgA was lowest in calves and highest in adults (Yin *et al.*, 2015). Free-ranging boar: IgA declined during weaning and increased again in older animals (Seo *et al.*, 2016). Free-ranging spotted hyenas: age-class specific variation was reported, with juveniles showing elevated IgA under high parasite pressure (Ferreira *et al.*, 2021; Veloso Soares *et al.*, 2025)
Sex	Generally absent; female-biased in some species	Free-ranging chimpanzees: faecal IgA did not differ between sexes (Lantz *et al.*, 2018). Captive lions: females exhibited higher faecal IgA than males (Serres-Corral *et al.*, 2025)
Reproduction/mating	Context-dependent; varies with reproductive state and species	Free-ranging red deer: gestation was associated with reduced total IgA, while lactation coincided with elevated parasite burden (Albery *et al.*, 2020). Free-ranging/provisioned Taihangshan macaques: adult females showed higher faecal IgA during the mating period than non-mating periods (Guo *et al.*, 2023). Free-ranging chimpanzees: faecal IgA did not differ by reproductive status (Lantz *et al.*, 2018)
Stress/HPA activation/glucocorticoids	Context-dependent; varies with stress type, timescale and species	Semi-free-ranging Barbary macaques: faecal IgA increased after ACTH-induced HPA activation and often covaried with fGCM, indicating acute stress-related immune activation (Behringer *et al.*, 2021). Captive Sichuan golden monkeys: IgA positively correlated with faecal cortisol seasonally (Huang *et al.*, 2014). Semi-domesticated/free-ranging reindeer: IgA showed negative association with faecal cortisol (Yin *et al.*, 2015). Captive Asian elephants: no consistent IgA–glucocorticoid relationship was observed (Kosaruk *et al.*, 2020)
Parasitism/pathogen exposure	Context-dependent; varies with parasite type, infection stage and host condition	Free-ranging spotted hyenas: IgA was positively associated with current parasite infections (Ferreira *et al.*, 2021). Mixed free-ranging/semi-managed African equids: IgA was positively correlated with strongyle FEC in individuals with good body condition, but not in nutritionally constrained animals (Tombak *et al.*, 2020). Free-ranging baboons: IgA was negatively associated with *Trichuris trichiura* egg counts and was not associated with parasite richness (Gesquiere *et al.*, 2020). Free-ranging red deer: lactation was linked to higher parasite egg counts with complex IgA patterns (Albery *et al.*, 2020). Free-ranging western chimpanzees: pathogen-specific IgA against *T. pallidum* was detectable in some individuals (Sereme *et al.*, 2024)
Illness/clinical gastrointestinal disturbance	Context-dependent; increased during localized gastrointestinal disturbance, reduced before acute systemic disease	Reintroduced/free-ranging Przewalski’s horses: IgA was higher in individuals with diarrhoea than in healthy controls (Li *et al.*, 2024). Captive Asian elephant calves: faecal IgA declined before onset of fatal EEHV-HD and preceded detectable viraemia (Boonprasert *et al.*, 2021)
Human activity/management conditions	No consistent directional association	Captive Asian elephants: faecal IgA did not differ consistently across tourist activity categories (Kosaruk *et al.*, 2020). Captive chimpanzees: IgA showed weak or inconsistent associations with behavioural welfare metrics (Hall *et al.*, 2021)
Seasonality/environment conditions	Frequently observed; context-dependent direction	Captive Sichuan golden monkeys: IgA varied seasonally and correlated with faecal cortisol (Huang *et al.*, 2014). Free-ranging chimpanzees: highest IgA concentrations occurred in the late dry season (Lantz *et al.*, 2018). Mixed free-ranging/semi-managed African equids: IgA–parasite associations differed by season (Tombak *et al.*, 2020)

Abbreviations: EEHV, elephant endotheliotropic herpesvirus; HD, haemorrhagic disease; FEC, faecal egg count; fGCM, faecal glucocorticoid metabolites.

In Asian elephants housed in tourism facilities, both faecal and salivary IgA varied across seasons and among camps differing in tourist density, human–animal interactions and management routines (Kosaruk *et al.*, 2020). Associations between IgA and glucocorticoids were inconsistent, and substantial inter-individual variability persisted even among elephants exposed to broadly similar conditions. Overall, elephant studies reported inconsistent IgA–glucocorticoid relationships and substantial inter-individual variability across management contexts.

Additional elephant studies reinforced this complexity. Multi-matrix assay development demonstrated pronounced baseline differences among individuals across faeces, saliva, urine and serum under standardized sampling conditions (Edwards *et al.*, 2019). Longitudinal monitoring of calves revealed overlapping IgA ranges in individuals with and without previous EEHV infection, alongside seasonal variation (Boonprasert *et al.*, 2021). In a single calf that later developed haemorrhagic disease, concurrent declines in faecal IgA and glucocorticoids preceded detectable viraemia, suggesting possible short-term sensitivity of mucosal immune markers to physiological destabilization. However, this observation was based on a single case and should be interpreted cautiously.

Zoo-based primate studies showed similar context-dependent patterns. In Barbary macaques, experimental activation of the HPA axis via ACTH challenge produced a concurrent increase in faecal IgA and glucocorticoid metabolites (Behringer *et al.*, 2021). Notably, this finding derives from one of the few studies incorporating biological validation, providing relatively strong support for acute IgA responsiveness under controlled physiological activation. In contrast, observational studies of professionally managed chimpanzees reported no consistent alignment between faecal IgA and behavioural diversity, stereotypy or glucocorticoid measures (Hall *et al.*, 2021). In these moderate-to-preliminary validation contexts, IgA responses appeared more variable and less predictably linked to welfare indices. At the same time, pathogen-specific faecal IgA remained detectable and corresponded with systemic antibody profiles in apes, supporting its value for infectious disease surveillance even when total IgA showed limited association with welfare-related measures (Yoshida *et al.*, 2016).

Data from captive carnivores were comparatively limited but broadly consistent with these patterns. In zoo-housed lions, faecal IgA was quantifiable following species-specific assay validation, yet concentrations showed no consistent directional relationship with management conditions or inferred stress (Serres-Corral *et al.*, 2025). As in elephants and primates, substantial inter-individual variability was evident, suggesting that baseline IgA levels are influenced by intrinsic physiological differences that may obscure subtle management effects.

Across captive mammal studies, acute physiological perturbation was associated with short-term increases in faecal IgA in the highest-validation evidence, whereas welfare- or management-associated patterns were more variable and largely observational. Inter-individual variation was prominent across elephants, chimpanzees and lions, limiting simple cross-sectional interpretation of faecal IgA in managed settings.

### Free-ranging mammals: ecological and disease drivers

As summarized in Table 4, studies in free-ranging mammals indicate that faecal IgA variation is influenced by ecological pressures including resource availability, social dynamics and sustained exposure to parasites and pathogens (Albery *et al.*, 2020; Ferreira *et al.*, 2021; Guo *et al.*, 2023). In wild baboons, faecal IgA remained detectable across repeated sampling despite environmental heterogeneity, demonstrating feasibility of field-based immune monitoring under non-standardized conditions (Gesquiere *et al.*, 2020).

A study of wild macaques further illustrated how ecological context may modulate mucosal immunity. In Taihangshan macaques, seasonal shifts in reproductive activity and social behaviour were associated with coordinated changes in faecal cortisol and IgA (Guo *et al.*, 2023). During the mating season, elevated cortisol coincided with lower IgA concentrations, whereas outside this period, associations weakened or shifted direction. These findings, derived from an observational field study without biological validation, indicate that IgA–cortisol relationships varied across reproductive periods in this free-ranging macaque population.

Large carnivore studies provided additional insight into ecological drivers of IgA. In spotted hyenas, faecal IgA concentrations were positively associated with current parasite infections (Ferreira *et al.*, 2021). More recent work linked IgA variation to gut microbiome composition in wild hyena clans (Veloso Soares *et al.*, 2025). These studies relied primarily on analytical validation without experimental immune perturbation, and therefore describe ecological association rather than experimentally confirmed immune responsiveness.

Ungulate studies provided complementary eco-immunological patterns. In red deer, total and parasite-specific IgA varied with reproductive state and parasite burden (Albery *et al.*, 2020). Gestation was associated with reduced total IgA, while lactation coincided with elevated parasite loads despite maintained mucosal IgA investment, suggesting potential trade-offs between reproductive effort and immune protection. In reindeer, seasonal oscillations in IgA, IgG and IgM did not closely mirror faecal cortisol patterns (Yin *et al.*, 2015), indicating that temporal variation in immunoglobulin profiles may not directly parallel endocrine dynamics under field conditions. These findings support interpretation of faecal IgA as an ecologically responsive mucosal marker, although effect sizes were generally modest relative to inter-individual variability.

Other herbivore studies demonstrated detectable parasite-associated IgA responses in less-characterized systems. In African equids, faecal IgA correlated with gastrointestinal nematode burdens (Tombak *et al.*, 2020), while in wild boar, cross-reactive ELISAs captured variation aligned with pathogen exposure (Seo *et al.*, 2016). As in several other moderate-to-preliminary validation contexts, these findings support ecological association but do not independently confirm mechanistic immune responsiveness.

Free-ranging primate studies also provided examples of pathogen-specific mucosal immunity. In western chimpanzees, faecal IgA targeting *T. pallidum* corresponded with systemic exposure patterns (Sereme *et al.*, 2024), and in bonobos, LCV-specific faecal IgA varied among regions (Yoshida *et al.*, 2016). These studies demonstrate feasibility of faecal-based serological surveillance in wild apes, although pathogen-specific applications depend heavily on prior assay validation.

Together, free-ranging studies most commonly linked faecal IgA variation with parasite exposure, reproductive or seasonal state and host-microbiome features, although most associations were observational and lacked biological validation.

### Cross-species comparative patterns

Cross-species comparison revealed three patterns that were not fully apparent from taxon-specific summaries alone. First, parasite- and pathogen-associated faecal IgA responses were most consistently reported in free-ranging systems. Positive associations with parasite exposure or parasite-specific immune activity were reported in spotted hyenas, red deer, African equids and wild boar (Seo *et al.*, 2016; Albery *et al.*, 2020; Tombak *et al.*, 2020; Ferreira *et al.*, 2021). These patterns were supported mainly by analytical validation rather than experimental immune perturbation, indicating ecological association rather than direct causal confirmation.

Second, IgA–stress relationships differed by timescale and validation depth. Experimental activation of the HPA axis in Barbary macaques produced concurrent increases in glucocorticoids and faecal IgA (Behringer *et al.*, 2021), whereas observational studies in macaques, elephants and chimpanzees reported weaker or inconsistent alignment between IgA and endocrine or behavioural indicators (Kosaruk *et al.*, 2020; Hall *et al.*, 2021; Guo *et al.*, 2023). Thus, acute physiological activation and chronic or ecologically complex stress contexts should not be interpreted as equivalent immune scenarios.

Third, direct numerical comparison across taxa was limited by species-specific baselines and methodological heterogeneity. Elephants, chimpanzees and lions showed pronounced inter-individual differences that were not fully explained by management or endocrine measures (Edwards *et al.*, 2019; Kosaruk *et al.*, 2020; Hall *et al.*, 2021; Serres-Corral *et al.*, 2025). Pathogen-specific applications, including LCV-specific IgA in bonobos and chimpanzees and *T. pallidum*-specific IgA in western chimpanzees, further demonstrated that faecal IgA can encode antigen-specific information when assays are appropriately adapted (Yoshida *et al.*, 2016; Sereme *et al.*, 2024).

Overall, cross-species inference was strongest for directional patterns within validated study systems, rather than for absolute faecal IgA concentrations across taxa. This comparative pattern supports cautious synthesis across species while preserving the distinct ecological and methodological context of each study.

## Discussion

This review shows that faecal IgA is measurable across diverse zoo and wild mammalian systems, but that its biological interpretation depends strongly on study context and validation depth. The clearest evidence for acute responsiveness comes from studies with experimental or physiological validation, whereas many associations with ecological exposure, life-history stage or management conditions derive from observational designs. This distinction is central to interpreting faecal IgA as a biological signal rather than as a uniform indicator of stress, welfare or health status.

The following sections develop this interpretation by presenting a conceptual framework, outlining methodological constraints, contrasting captive and wild contexts and identifying priorities for future research.

### Conceptual model: how faecal IgA reflects mammalian health in zoo and wild settings

**Figure 2:**
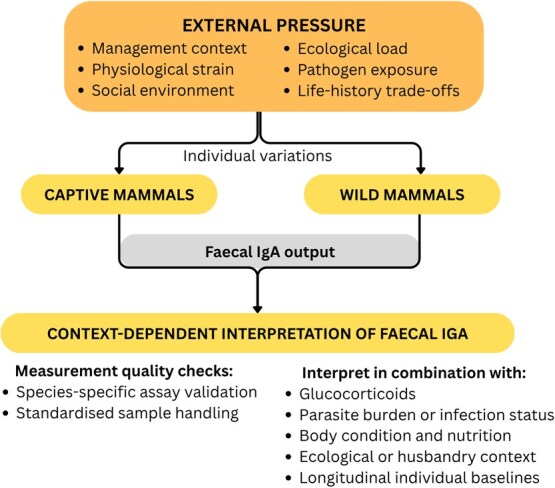
Conceptual framework for interpreting faecal IgA as a non-invasive indicator of mucosal immunity and health in zoo and wild mammals.

The synthesis supports an interpretive framework in which faecal IgA reflects mucosal immune allocation shaped by external pressures, host physiological state and measurement quality. Rather than treating faecal IgA as a direct readout of welfare or stress, the framework links four elements: external ecological or management pressures, mucosal immune dynamics, complementary biomarkers and downstream health or welfare outcomes (Fig. 2). This framework is intended as an interpretive structure rather than a mechanistic or causal model.

A first component concerns external pressures. In zoo and wild settings, mammals experience combinations of ecological and anthropogenic challenges, including food availability, reproductive effort, social competition, pathogen exposure and human interaction. These pressures may alter antigenic exposure at the gut mucosa and interact with broader physiological regulation. Observational studies in macaques and reindeer illustrate this principle, although causal pathways remain to be experimentally tested (Yin *et al.*, 2015; Guo *et al.*, 2023).

A second component involves the mucosal immune response itself. Faecal IgA may increase with microbial exposure or gastrointestinal challenge, but immune allocation may be constrained under energetic limitation or prolonged physiological strain. Across the reviewed studies, parasite-associated IgA responses were reported in ungulates and carnivores, whereas reduced IgA was observed in some primate contexts during energetically demanding periods (Albery *et al.*, 2020; Gesquiere *et al.*, 2020; Tombak *et al.*, 2020; Ferreira *et al.*, 2021; Guo *et al.*, 2023). These patterns are consistent with immune trade-off frameworks, in which immune investment is constrained by energetic, life-history and environmental demands (Sheldon and Verhulst, 1996; Lochmiller and Deerenberg, 2000; Schmid-Hempel, 2003; Schoenle *et al.*, 2018), although most evidence derives from correlational designs rather than direct manipulation of energetic or immune pathways.

A third dimension concerns integration with complementary biomarkers. Glucocorticoids index endocrine activation across diverse stimuli, whereas faecal IgA reflects mucosal immune processes that may not parallel endocrine change. Across captive elephants, chimpanzees and lions, IgA showed inconsistent alignment with glucocorticoid or behavioural measures, supporting its interpretation as complementary rather than redundant information (Kosaruk *et al.*, 2020; Hall *et al.*, 2021; Serres-Corral *et al.*, 2025).

A fourth dimension links mucosal immune patterns to health outcomes. Elevated faecal IgA may indicate immune engagement, antigenic challenge or gastrointestinal disturbance (Edwards *et al.*, 2019; Ferreira *et al.*, 2021), whereas low or declining IgA may reflect reduced mucosal investment in some contexts (Boonprasert *et al.*, 2021; Guo *et al.*, 2023). Thus, direction alone is insufficient for interpretation. Biological meaning depends on whether the underlying driver is protective immune activity, pathogen exposure, clinical disturbance or physiological strain.

Taken together, this framework therefore emphasizes interpretation of faecal IgA in relation to ecological context, energetic capacity, validation strength and complementary physiological or clinical information. Its application depends on species-specific assay validation and standardized sample handling to minimize methodological artefacts.

### Methodological limitations and challenges

Application of faecal IgA as a biomarker in mammalian health assessment requires careful methodological consideration, as relatively small differences in assay performance, sample handling and biological context can generate substantial variation in measured concentrations. Several technical and biological factors therefore constrain interpretation.

A primary challenge involves antibody specificity and cross-reactivity. Many non-invasive IgA studies employ polyclonal antibodies developed against human or domestic animal IgA because conserved IgA epitopes permit cross-reactivity across mammals. This approach has yielded biologically interpretable results in elephants, primates, carnivores and equids when paired with appropriate standards and validation procedures (Edwards *et al.*, 2019; Kosaruk *et al.*, 2020; Ferreira *et al.*, 2021; Serres-Corral *et al.*, 2025). However, cross-reactivity cannot be assumed across taxa, as IgA structure, subclass distribution and glycosylation patterns vary among species (Seo *et al.*, 2016). Assays performing reliably in one lineage may exhibit altered sensitivity or specificity in another. Accordingly, studies reporting robust performance consistently included empirical validation procedures, most commonly parallelism and dilution testing relative to standard curves (Edwards *et al.*, 2019; Pethig *et al.*, 2024). Without such validation, apparent biological differences may instead reflect assay artefacts. These requirements align with broader immunoassay standards emphasizing matrix-specific validation and transparent reporting (Andreasson *et al.*, 2015; Hickford *et al.*, 2023), reinforcing this as a general best practice.

Sample preservation and extraction represent a second major source of variability. Secretory IgA and related antibody proteins are susceptible to degradation after excretion, particularly in faecal matrices rich in proteases. Across the reviewed literature, rapid freezing or lyophilization was consistently associated with more reliable IgA quantification (Edwards *et al.*, 2019; Pethig *et al.*, 2024). Experimental evaluations demonstrated measurable loss of IgA under ambient storage, whereas samples maintained under consistent cold-chain conditions retained detectability (Pethig *et al.*, 2024). Similar preservation approaches were employed successfully in elephants, primates and carnivores (Lantz *et al.*, 2018; Kosaruk *et al.*, 2020; Serres-Corral *et al.*, 2025). Although extraction buffers and processing protocols differed among laboratories, prompt stabilization appeared more influential than specific buffer composition. Nonetheless, differences in extraction efficiency across matrices and institutions may limit direct quantitative comparison, underscoring the need for procedural harmonization.

Analytical validation forms a third methodological pillar. Studies reporting clearer biological patterns generally documented assay performance metrics such as parallelism, spike-and-recovery testing, sensitivity thresholds and acceptable intra- and inter-assay CVs. In elephants, recovery of exogenous IgA approached expected values, and CVs were within commonly accepted ranges (Edwards *et al.*, 2019; Kosaruk *et al.*, 2020). Comparable validation procedures were reported in lemurs, hyenas, equids and lions (Tombak *et al.*, 2020; Ferreira *et al.*, 2021; Pethig *et al.*, 2024; Serres-Corral *et al.*, 2025). By contrast, studies relying primarily on previously validated assays without re-testing in the focal population often reported greater variability or more ambiguous associations, although direct comparison across validation tiers remains limited. Together, these findings support the need for transparent reporting and species-specific assay confirmation.

Beyond technical factors, faecal IgA is influenced by biological variables that can confound interpretation if not explicitly incorporated into study design. Age-related effects were among the most consistently reported patterns. In chimpanzees, musk deer and reindeer, faecal IgA varied across developmental stages, consistent with maturation and later-life modulation of mucosal immunity (Yin *et al.*, 2015; Lantz *et al.*, 2018; Li *et al.*, 2021). Sex differences were detected in some taxa, including reindeer and lions, although not uniformly across species (Yin *et al.*, 2015; Lantz *et al.*, 2018; Serres-Corral *et al.*, 2025). Nutritional condition further modulated IgA expression. In African equids, individuals in better body condition exhibited stronger IgA responses to parasite challenge despite comparable burdens (Tombak *et al.*, 2020), consistent with eco-immunological frameworks linking immune allocation to energetic capacity (Albery *et al.*, 2020). These findings highlight the need to model demographic and condition variables explicitly.

Parasitism and disease exposure represent additional drivers of variation. In multiple species, elevated faecal IgA was associated with parasite challenge, reflecting immune activation rather than improved health status (Tombak *et al.*, 2020; Ferreira *et al.*, 2021). Such associations should therefore be interpreted as immune activation rather than improved condition, particularly in parasite-exposed populations. Seasonal oscillations in IgA were documented in free-ranging primates and ungulates, and diurnal patterns were observed in elephants and chimpanzees (Lantz *et al.*, 2018; Kosaruk *et al.*, 2020; Plangsangmas *et al.*, 2020). These temporal dynamics emphasize the importance of standardized sampling schedules and repeated-measures designs.

Finally, substantial intra- and inter-individual heterogeneity complicates cross-sectional interpretation. Within populations, faecal IgA concentrations often varied markedly among individuals under similar environmental conditions (Edwards *et al.*, 2019; Serres-Corral *et al.*, 2025). Absolute values may therefore be less informative than within-individual trajectories over time. Longitudinal designs establishing individual baselines therefore provide more robust interpretation than single time-point comparisons, consistent with broader best practices in non-invasive immunological research.

In summary, interpretation of faecal IgA depends heavily on methodological rigour. Assay validation, standardized sample handling and explicit modelling of demographic and ecological covariates are essential for distinguishing biological signal from methodological artefacts. These considerations are particularly important when comparing results across taxa, institutions or ecological contexts.

### Captive vs wild differences in IgA interpretation

Captive and free-ranging mammals inhabit distinct immunological environments shaped by differences in pathogen exposure, resource stability, social structure and management intensity. In zoological settings, immune dynamics are influenced by controlled diets, structured social groups, enclosure design, enrichment regimes, veterinary intervention and predictable human interaction. In contrast, wild mammals often experience fluctuating parasite exposure, seasonal resource limitation, climatic stressors and life-history trade-offs that impose variable immune demands. Because mucosal immunity reflects allocation of finite energetic resources to competing physiological processes, similar changes in faecal IgA concentration may carry different biological implications depending on ecological context. This perspective is consistent with broader literature indicating that chronic strain can suppress mucosal IgA under some conditions, whereas stable or less disruptive environments may support more consistent IgA profiles (Staley *et al.*, 2018), although direct experimental confirmation in wildlife remains limited.

In captive systems, faecal IgA has increasingly been incorporated into welfare assessment alongside endocrine and behavioural measures. Under relatively stable nutritional and parasitological conditions, sustained physiological strain may be associated with reduced mucosal immune investment. Horses undergoing hospitalization and surgical procedures exhibited declines in faecal IgA concurrent with elevated glucocorticoids (May *et al.*, 2024), and IgA has been applied alongside endocrine and behavioural indicators in zoo-housed carnivores and primates (Hall *et al.*, 2021; Serres-Corral *et al.*, 2025). In these settings, persistently low IgA may suggest reduced mucosal immune investment, although thresholds remain species specific and rarely standardized.

Captive systems also show that IgA responses are not unidirectional. In tourism-managed Asian elephants, faecal IgA differed among camps in patterns not fully explained by tourist density or riding modality (Kosaruk *et al.*, 2020). Inter-individual variability was substantial, and IgA did not consistently align with glucocorticoid profiles or simple management descriptors. Similar heterogeneity has been reported in other captive populations (Moresco *et al.*, 2022). Elevated IgA in captivity may therefore reflect immune activation rather than improved condition, as reported in clinically affected elephants and horses with gastrointestinal pathology (Edwards *et al.*, 2019; Żak-Bochenek *et al.*, 2025). These findings indicate that elevated IgA in managed settings may signal either adaptive immune engagement or acute challenge.

In free-ranging mammals, faecal IgA more frequently aligns with ecological immune demands. Because free-ranging animals often encounter variable parasite exposure, microbial diversity and nutritional constraints, elevated IgA may reflect antigenic load rather than favourable condition. For example, juvenile spotted hyenas with parasite-associated IgA and mucin responses showed reduced survival, suggesting that high IgA can reflect costly immune activation (Ferreira *et al.*, 2021). Comparable parasite-associated patterns were reported in African equids, particularly among individuals in better condition (Tombak *et al.*, 2020).

Stress-associated reductions in IgA may also occur in free-ranging contexts, but they are difficult to separate from concurrent ecological changes. In Taihangshan macaques, mating-season social competition coincided with elevated cortisol and reduced faecal IgA (Guo *et al.*, 2023), consistent with short-term reallocation of resources away from mucosal immunity. Seasonal variation in wild chimpanzees further illustrates temporal variation in IgA under natural conditions (Lantz *et al.*, 2018). These patterns arise within complex ecological matrices, making it difficult to isolate stress effects from concurrent changes in pathogen exposure or nutritional status.

Across both managed and free-ranging settings, interpretation is limited by inter-individual variability and the predominance of observational designs. Captive–wild contrasts should therefore be viewed as probabilistic and context specific rather than mechanistically established across taxa (Staley *et al.*, 2018; Whitham *et al.*, 2023). The principal divergence between managed and free-ranging settings lies in interpretation of elevated IgA. In managed systems with relatively low parasite exposure, moderate IgA increases may reflect maintained mucosal defence or short-term immune stimulation. In free-ranging populations subject to higher antigenic load, elevated IgA more often accompanies parasite challenge or gastrointestinal disturbance (Ferreira *et al.*, 2021). These contrasts reinforce that faecal IgA is not intrinsically indicative of positive or negative condition.

### Research gaps and future directions

Although faecal IgA shows strong potential as a non-invasive indicator of mucosal immunity, its application in conservation physiology remains at an early stage. Current evidence captures only a limited portion of the taxonomic, ecological and methodological diversity required for broad inference. Recent syntheses of non-invasive biomarkers have similarly highlighted that limited taxonomic breadth and inconsistent study design constrain broader ecological inference (Schilling *et al.*, 2022).

A major priority is expansion of taxonomic coverage. Existing studies are concentrated in primates, a limited number of ungulates and Asian elephants, reflecting logistical accessibility more than mammalian diversity (Lantz *et al.*, 2018; Kosaruk *et al.*, 2020; Li *et al.*, 2021). Many carnivore lineages, small-bodied mammals and marine taxa remain unexamined. Recent assay validation in lions demonstrates feasibility in previously unstudied taxa but also illustrates how limited current taxonomic coverage remains (Serres-Corral *et al.*, 2025). Broader comparative sampling will therefore be necessary to determine whether observed IgA patterns reflect conserved mammalian principles or lineage-specific immune strategies shaped by digestive physiology, microbiome structure and exposure history.

A second challenge is the lack of coordinated methodological frameworks that support cross-study comparison and applied use. Future progress will depend not only on identifying technical limitations, but also on establishing harmonized validation, normalization and reporting standards. Such standardization would facilitate meta-analysis, longitudinal monitoring and multi-institutional comparison across taxa and ecological settings.

Faecal IgA also remains underutilized within integrative physiological designs. Interpretation generally improves when faecal IgA is evaluated alongside endocrine, parasitological, microbiome or clinical measures rather than as a standalone endpoint (Kosaruk *et al.*, 2020; Ferreira *et al.*, 2021; Guo *et al.*, 2023). Future research should prioritize integrative physiological designs that model faecal IgA within coordinated immune–endocrine–energetic networks.

Experimental evidence also remains limited. Most available studies are observational, restricting causal inference regarding the drivers and consequences of IgA variation. Experimental or quasi-experimental approaches, including enrichment interventions, dietary manipulations, vaccination programmes or controlled physiological challenges, would provide stronger evidence of how specific pressures influence mucosal IgA dynamics (Behringer *et al.*, 2021; Boonprasert *et al.*, 2021). Without such approaches, distinguishing adaptive immune engagement from maladaptive immune strain will remain difficult.

Long-term monitoring frameworks are similarly scarce. Because faecal IgA varies with age, season, infection history and individual immune capacity, single measurements remain inherently ambiguous. Individual baselines and population-level reference intervals have proven informative in chimpanzees and other taxa (Whitham *et al.*, 2023; Pethig *et al.*, 2024), enabling interpretation based on deviation from baseline rather than absolute concentrations. Embedding faecal IgA into long-term zoo welfare programmes or field demographic studies may therefore improve detection of gradual immune shifts preceding overt disease or demographic change.

Finally, translation into practical conservation and welfare management remains limited. Despite growing empirical support, faecal IgA is rarely incorporated into routine monitoring or decision-making frameworks. Validated assays and feasible sampling protocols already exist for several taxa, including elephants, chimpanzees, lemurs and lions (Edwards *et al.*, 2019; Kosaruk *et al.*, 2020; Pethig *et al.*, 2024; Serres-Corral *et al.*, 2025). Future implementation will require collaboration among researchers, veterinarians and animal managers to establish interpretation frameworks, decision thresholds and pilot monitoring programmes that demonstrate practical utility.

Together, these priorities define the next stage of faecal IgA research: broader taxonomic coverage, harmonized methodological standards, integrative physiological study designs and long-term monitoring frameworks. Addressing these gaps will determine whether faecal IgA can move from a promising research metric to a robust applied tool within conservation physiology.

## Conclusion

Faecal IgA is a technically feasible and biologically informative marker of mucosal immune activity in zoo and wild mammals. Across the reviewed studies, its strongest support lies in detecting mucosal immune engagement and acute physiological responsiveness, whereas many associations with welfare, chronic stress and ecological exposure remain observational and context dependent. Interpretation therefore depends on species biology, ecological setting and validation depth.

Rather than serving as a standalone indicator of stress or welfare, faecal IgA is best used as a complementary measure within longitudinal health assessment frameworks. Future work should prioritize species-specific assay validation, harmonized sample handling, broader taxonomic coverage and experimentally informed study designs. With these advances, faecal IgA can contribute meaningfully to non-invasive conservation physiology, particularly in systems where repeated sampling is essential and invasive monitoring is impractical.

## Supplementary Material

Web_Material_coag054

## Data Availability

All data for this study are included in this article (and its supplementary information files).
