# Supplementary material for: Faecal immunoglobulin A as a non-invasive biomarker of mucosal immunity and health in zoo and wild mammals
Source: Conserv Physiol. 2026 Jul 28;14(1):coag054. doi: 10.1093/conphys/coag054 (PMC13411278; doi:10.1093/conphys/coag054)
Supplement: Web_Material_coag054 [file web_material_coag054.zip › Appendix S1.docx]

**Appendix S1.**

**A. Full database-specific search strategies**

**PubMed:** (“immunoglobulin A” OR IgA OR “secretory IgA” OR sIgA) AND (elephant OR tiger OR lion OR leopard OR panthera OR macaque OR baboon OR chimpanzee OR gorilla OR primate OR lemur OR deer OR reindeer OR musk deer OR giraffe OR zebra OR equid OR ungulate OR carnivore OR bear OR canid OR zoo OR wildlife OR sanctuary OR captive). **Scopus:** TITLE-ABS-KEY(“immunoglobulin A” OR IgA OR “secretory IgA” OR sIgA) AND TITLE-ABS-KEY(elephant OR tiger OR lion OR leopard OR panthera OR macaque OR baboon OR chimpanzee OR gorilla OR primate OR lemur OR deer OR reindeer OR “musk deer” OR giraffe OR zebra OR equid OR ungulate OR carnivore OR bear OR canid OR zoo OR wildlife OR sanctuary OR captive) AND PUBYEAR > 2014. **Embase:** (‘immunoglobulin a’:ti,ab OR iga:ti,ab OR ‘secretory iga’:ti,ab OR siga:ti,ab) AND (elephant:ti,ab OR tiger:ti,ab OR lion:ti,ab OR leopard:ti,ab OR panthera:ti,ab OR macaque:ti,ab OR baboon:ti,ab OR chimpanzee:ti,ab OR gorilla:ti,ab OR primate:ti,ab OR lemur:ti,ab OR deer:ti,ab OR ‘musk deer’:ti,ab OR reindeer:ti,ab OR giraffe:ti,ab OR zebra:ti,ab OR equid:ti,ab OR ungulate:ti,ab OR carnivore:ti,ab OR bear:ti,ab OR canid:ti,ab OR zoo:ti,ab OR wildlife:ti,ab OR sanctuary:ti,ab OR captive:ti,ab) AND [2015-2025]/py.

**Google Scholar:** "fecal IgA" OR "immunoglobulin A" (wildlife OR zoo OR captive OR "free-ranging" OR sanctuary) mammal

**B. Rule-based title pre-screening terms (used only for removal of clearly ineligible records)**
Records were flagged for exclusion if the title contained one or more of the following term groups:

- **Human clinical populations:** “human”, “patient”, “children”, “infant”, “elderly”
- **Laboratory rodents:** “mouse”, “mice”, “rat”, “murine”, “murinae”
- **Domestic livestock / companion animals:** “swine”, “pig”, “porcine”, “cattle”, “bovine”, “cow”, “calf”, “chicken”, “poultry”, “avian”, “dog”, “canine”, “cat”, “feline”, “horse”
- **Aquatic / fish models:** “fish”, “zebrafish”, “aquatic”
- **In vitro / molecular engineering:** “in vitro”, “cell line”, “receptor”, “binding assay”, “transfection”, “clone”, “engineered”, “synthetic”
- **Irrelevant biomedical conditions (title-level):** “celiac disease”, “coeliac disease”, “nephropathy”, “Berger”, “vasculitis”
- **Non-primary formats:** “review”
